# Supplementary material for: Isolation of Yeast and LAB from Dry Coffee Pulp and Monitoring of Organic Acids in Inoculated Green Beans
Source: Foods. 2023 Jul 6;12(13):2622. doi: 10.3390/foods12132622 (PMC10340337; doi:10.3390/foods12132622)

Supplementary Table S1. Organic acids quantified in the fermented coffee beans with different yeast isolates

Acids/ time (h)	Treatments Y1R	Y5R	Y10W	Y12aR	Y12bR	Y18W	Y19W	Y38W	Y43W	Control
Acetic										
24	0.55±0.01*	0.46±0.01	0.51±0.04	0.49±0.01	0.54±0.10*	0.47±0.01	0.50±0.01	0.53±0.01	0.47±0.00	0.45±0.01
48	0.52±0.01	0.50±0.03	0.53±0.01	0.58±0.02*	0.67±0.05***	0.53±0.02	0.57±0.02*	0.60±0.05**	0.58±0.06*	0.47±0.02
72	1.13±0.01***	0.56±0.04*	0.61±0.02**	0.60±0.03**	1.33±0.28***	0.58±0.03**	0.62±0.03***	0.60±0.02**	0.64±0.05***	0.46±0.02
Citric										
24	0.41±0.03**	0.51±0.03	0.58±0.13	0.48±0.08*	0.58±0.15	0.64±0.05	0.49±0.05	0.53±0.10	0.45±0.03*	0.61±0.21
48	0.38±0.01**	0.63±0.02	0.47±0.04	0.82±0.22***	0.47±0.04	0.54±0.07	0.49±0.05	0.52±0.05	0.55±0.05	0.57±0.18
72	0.14±0.00***	0.45±0.12***	0.45±0.03***	0.48±0.11***	0.22±0.02***	0.42±0.04***	0.45±0.13***	0.45±0.07***	0.44±0.02***	0.72±0.19
Formic										
24	0.42±0.02***	0.45±0.01***	0.44±0.02***	0.44±0.01***	0.43±0.03***	0.46±0.01**	0.42±0.01***	0.45±0.03***	0.45±0.02***	0.49±0.05
48	0.43±0.01	0.45±0.01	0.40±0.00***	0.41±0.00***	0.40±0.00***	0.45±0.00	0.40±0.00***	0.40±0.00***	0.43±0.02	0.45±0.01
72	0.40±0.01	0.41±0.01	0.40±0.00	0.40±0.00	0.40±0.00	0.40±0.00	0.40±0.00	0.40±0.00	0.40±0.00	0.42±0.02
Lactic										
24	0.29±0.02	0.30±0.01	0.31±0.01	0.29±0.00	0.45±0.11	0.33±0.03	0.30±0.01	0.29±0.01	0.41±0.04	0.40±0.11
48	0.44±0.02	0.30±0.00	0.29±0.01	0.48±0.12	0.31±0.03	0.39±0.12	0.33±0.06	0.29±0.01	0.45±0.11	0.37±0.12
72	2.41±0.03***	0.30±0.01	0.30±0.01	0.41±0.09	2.21±0.78***	0.40±0.13	0.30±0.00	0.31±0.07	0.39±0.06	0.34±0.04
Malic										
24	0.45±0.03	0.47±0.02	0.55±0.10**	0.45±0.03	0.54±0.06**	0.54±0.01**	0.42±0.00	0.49±0.06	0.41±0.05	0.43±0.04
48	0.33±0.01***	0.52±0.01	0.43±0.04	0.57±0.07**	0.43±0.04	0.50±0.05	0.48±0.02	0.48±0.02	0.45±0.05	0.48±0.05
72	0.27±0.01***	0.41±0.03	0.46±0.03	0.45±0.06	0.30±0.10***	0.35±0.05*	0.45±0.05	0.44±0.01	0.38±0.07*	0.47±0.11
Quinic										
24	1.15±0.07*	1.49±0.18***	1.38±0.09**	1.44±0.16***	1.91±0.23***	1.76±0.35***	1.18±0.14*	1.57±0.43***	1.32±0.11**	0.74±0.27
48	1.25±0.09*	1.83±0.09	1.25±0.16*	1.43±0.14	2.36±0.65**	1.75±0.40	1.52±0.40	1.64±0.32	1.74±0.03	1.73±0.41
72	0.50±0.02***	1.26±0.32**	1.50±0.12	1.49±0.38	0.49±0.19***	1.76±0.10	1.12±0.14***	1.29±0.15*	1.37±0.20*	1.88±0.70
Succinic										
24	0.24±0.01***	0.23±0.00***	0.24±0.01***	0.24±0.01***	0.24±0.01***	0.23±0.00***	0.25±0.00***	0.25±0.00***	0.23±0.00***	0.38±0.13
48	0.24±0.01	0.25±0.00	0.24±0.01	0.27±0.00	0.25±0.00	0.25±0.01	0.25±0.00	0.26±0.01	0.25±0.01	0.23±0.00
72	0.28±0.03	0.24±0.01	0.26±0.01	0.25±0.01	0.29±0.02*	0.24±0.01	0.25±0.01	0.25±0.01	0.25±0.01	0.25±0.03
Total										
24	3.51±0.12	3.91±0.23	4.00±0.36	3.82±0.21	4.70±0.44***	4.43±0.29**	3.56±0.19	4.11±0.57*	3.74±0.17	3.51±0.16
48	3.59±0.13*	4.47±0.13	3.62±0.22*	4.55±0.27	4.90±0.64*	4.43±0.59	4.03±0.53	4.21±0.43	4.46±0.15	4.29±0.77
72	5.14±0.07	3.62±0.50**	3.97±0.18	4.08±0.67	5.25±0.85*	3.60±0.14	3.89±0.69**	4.00±0.22*	3.88±0.16*	4.54±0.95
Average	4.08±0.03	4.00±0.19	3.86±0.10	4.15±0.25	4.95±0.20*	4.15±0.23	3.83±0.26	4.11±0.17	4.03±0.01	4.11±0.42

Results are presented as mean ± SD ($n = 4$) of organic acids (mg/g). For each row, concentration denoted with star labels are significantly different from control (* for $p < .05$, ** for $p < .01$, *** for $p < .001$)

Supplementary Table S2. Organic acids quantified in the fermented coffee beans with different lactic acid bacteria isolates

Acids	Time (h)	Treatments											
		L4	L5	L6	L6b	L8	L10	L12	L14	L16	L17	L17b	Control
Acetic	12	0.47±0.01	0.48±0.01	0.46±0.01	0.47±0.01	0.47±0.01	0.48±0.03	0.49±0.01	0.49±0.02	0.46±0.01	0.47±0.01	0.47±0.01	0.47±0.01
	24	0.48±0.01	0.48±0.02	0.46±0.00	0.47±0.01	0.48±0.02	0.48±0.03*	0.47±0.00	0.49±0.01*	0.47±0.01	0.47±0.01	0.45±0.01	0.45±0.00
	48	0.47±0.00	0.46±0.01	0.48±0.02	0.45±0.00	0.47±0.01	0.47±0.01	0.46±0.01	0.44±0.01	0.52±0.11**	0.47±0.01	0.49±0.01*	0.45±0.01
	72	0.48±0.01	0.45±0.01	0.47±0.00	0.46±0.01	0.47±0.01	0.46±0.02	0.47±0.01	0.46±0.02	0.46±0.00	0.46±0.02	0.49±0.02	0.46±0.00
Citric	12	0.78±0.23	0.55±0.07***	0.52±0.07***	0.57±0.01***	0.55±0.04***	0.73±0.06	0.63±0.09*	0.68±0.04	0.50±0.01***	0.71±0.10	0.55±0.02***	0.79±0.01
	24	0.54±0.05	0.47±0.03	0.45±0.03	0.48±0.04	0.52±0.04	1.09±0.10***	0.81±0.08***	0.56±0.03	0.62±0.01*	0.43±0.00	0.49±0.01	0.47±0.03
	48	0.50±0.01	0.39±0.03	0.47±0.03	0.30±0.07*	0.50±0.05	0.48±0.04	0.47±0.02	0.34±0.02*	0.49±0.01	0.46±0.02	0.5±0.01	0.46±0.02
	72	0.86±0.20***	0.77±0.14**	0.51±0.15	0.75±0.29*	0.73±0.14	0.88±0.06***	0.80±0.22**	0.63±0.01	0.69±0.01	0.59±0.01	0.66±0.00	0.61±0.03
Formic	12	0.44±0.01	0.44±0.00	0.44±0.01	0.44±0.02	0.44±0.02	0.46±0.01*	0.45±0.01	0.45±0.02	0.41±0.01***	0.45±0.01	0.42±0.02*	0.44±0.01
	24	0.46±0.01***	0.45±0.02***	0.44±0.01	0.43±0.01	0.44±0.01	0.45±0.01**	0.44±0.02*	0.45±0.02***	0.44±0.01*	0.44±0.01*	0.42±0.01	0.42±0.01
	48	0.44±0.02	0.44±0.01	0.45±0.01	0.41±0.01***	0.46±0.01	0.46±0.00	0.43±0.01*	0.44±0.00	0.46±0.01	0.47±0.00*	0.43±0.01*	0.45±0.00
	72	0.46±0.01***	0.41±0.01	0.42±0.02	0.42±0.01	0.47±0.01***	0.45±0.01**	0.45±0.01***	0.44±0.01*	0.48±0.01***	0.43±0.01	0.48±0.01***	0.42±0.01
Lactic	12	0.34±0.01	0.44±0.02***	0.32±0.02	0.30±0.01	0.48±0.03***	0.46±0.03***	0.47±0.06***	0.39±0.03**	0.31±0.01	0.40±0.02***	0.34±0.02	0.31±0.01
	24	0.30±0.02	0.31±0.01	0.30±0.01	0.33±0.02	0.32±0.03	0.34±0.02	0.35±0.02	0.37±0.01*	0.44±0.01***	0.30±0.01	0.34±0.02	0.32±0.01
	48	0.31±0.02	0.32±0.02	0.33±0.01	0.32±0.02	0.41±0.10***	0.33±0.01	0.38±0.05**	0.33±0.03	0.40±0.04***	0.37±0.02**	0.40±0.03***	0.30±0.00
	72	0.33±0.01	0.32±0.02	0.42±0.12**	0.37±0.06	0.33±0.02	0.35±0.02	0.41±0.05**	0.42±0.02**	0.49±0.01***	0.33±0.01	0.43±0.04***	0.33±0.06
Malic	12	0.51±0.06	0.40±0.02***	0.47±0.03	0.47±0.02	0.42±0.02***	0.47±0.03	0.44±0.02**	0.51±0.00	0.40±0.01***	0.47±0.00	0.43±0.02**	0.51±0.01
	24	0.48±0.03	0.45±0.04	0.45±0.03	0.45±0.02	0.48±0.03	0.49±0.04	0.43±0.04	0.48±0.01	0.47±0.01	0.40±0.01*	0.50±0.00	0.46±0.03
	48	0.45±0.02	0.38±0.02***	0.48±0.03	0.39±0.01***	0.45±0.01	0.50±0.01	0.43±0.02*	0.39±0.02***	0.42±0.01*	0.46±0.01	0.54±0.01*	0.48±0.01
	72	0.60±0.05*	0.53±0.06	0.47±0.10**	0.55±0.10	0.53±0.05	0.63±0.03***	0.55±0.08	0.56±0.01	0.54±0.01	0.49±0.02*	0.63±0.01***	0.54±0.01
Quinic	12	1.89±0.71***	1.25±0.17	1.24±0.17	1.24±0.11	1.13±0.12	1.40±0.20	1.46±0.18	1.52±0.07	0.95±0.04	1.42±0.06	1.18±0.1	1.23±0.05
	24	1.73±0.18*	1.26±0.09	1.31±0.16	1.27±0.20	1.61±0.16	1.41±0.22	1.37±0.15	1.37±0.03	1.19±0.50	1.11±0.02	1.38±0.05	1.24±0.08
	48	1.65±0.12	1.05±0.15*	1.50±0.20	0.96±0.05**	1.46±0.07	1.40±0.05	1.44±0.05	0.93±0.03**	1.29±0.06	1.51±0.02	1.61±0.09	1.55±0.01
	72	2.85±0.43***	2.26±0.60*	1.91±0.50	2.09±0.72	2.16±0.44	2.26±0.32*	2.51±0.64***	1.74±0.50	2.14±0.04	1.87±0.11	2.13±0.09	1.81±0.15
Succinic	12	0.23±0.00	0.23±0.00	0.23±0.00	0.22±0.00	0.22±0.00	0.23±0.00	0.23±0.00	0.24±0.00	0.23±0.00	0.23±0.01	0.22±0.00	0.22±0.00
	24	0.22±0.02	0.23±0.00	0.22±0.00	0.22±0.00	0.24±0.01	0.23±0.01	0.23±0.00	0.23±0.01	0.23±0.01	0.23±0.00	0.24±0.01	0.23±0.01
	48	0.23±0.01	0.22±0.01	0.23±0.01	0.23±0.01	0.23±0.01	0.23±0.01	0.24±0.00	0.22±0.00	0.23±0.01	0.22±0.00	0.23±0.00	0.23±0.00
	72	0.24±0.01	0.23±0.01	0.23±0.00	0.25±0.03	0.25±0.02	0.24±0.01	0.24±0.01	0.25±0.01	0.24±0.00	0.23±0.01	0.24±0.00	0.24±0.01
Total	12	4.65±0.98**	3.78±0.28	3.69±0.28	3.70±0.15	3.72±0.23	4.22±0.30	4.16±0.35	4.26±0.05	3.14±0.16*	4.10±0.11	3.56±0.08	3.83±0.29
	24	4.20±0.27**	3.65±0.15	3.64±0.19	3.66±0.28	4.08±0.25*	4.49±0.36***	4.10±0.24*	3.94±0.04	3.80±0.47	3.31±0.12	3.82±0.07	3.51±0.27
	48	4.04±0.15	3.26±0.18*	3.93±0.25	3.07±0.11**	3.98±0.18	3.87±0.06	3.85±0.04	3.10±0.02**	3.80±0.18	3.92±0.07	4.14±0.09	3.93±0.00
	72	5.82±0.66***	4.96±0.84*	4.43±0.81	4.89±1.20*	4.93±0.64*	5.27±0.45***	5.43±0.99***	4.49±0.49	5.05±0.06**	4.40±0.14	5.06±0.08**	4.22±0.29
Average		4.68±0.90	3.91±0.77	3.92±0.52	3.83±0.88	4.18±0.58	4.46±0.61	4.39±0.80	3.95±0.59	3.95±0.75	3.93±0.43	4.14±0.59	3.87±0.34

Results are presented as mean ± SD (n = 4) of organic acids (mg/g). For each row, concentration denoted with star labels are significantly different from control (* for $p < .05$, ** for $p < .01$, *** for $p < .001$)

Supplementary Figure S1. Flow chart of fermentation process in section 2.4

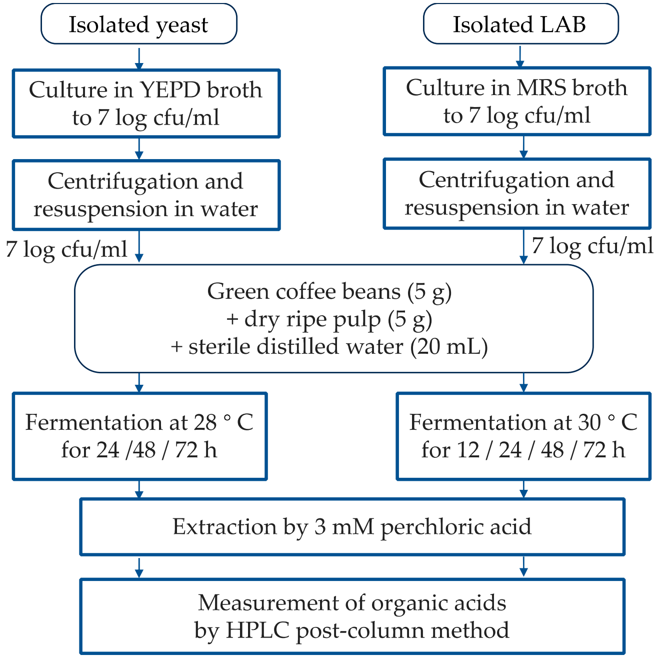

Supplement: Supplementary file 1 [file foods-12-02622-s001.zip › foods-2476622-supplementary.pdf]
